# Unraveling the Influence of Land-Use Change on δ^13^C, δ^15^N, and Soil Nutritional Status in Coniferous, Broadleaved, and Mixed Forests in Southern China: A Field Investigation

**DOI:** 10.3390/plants10081499

**Published:** 2021-07-21

**Authors:** Taimoor Hassan Farooq, Xiaoyong Chen, Awais Shakoor, Yong Li, Jun Wang, Muhammad Haroon U. Rashid, Uttam Kumar, Wende Yan

**Affiliations:** 1 Bangor College China, a Joint Unit of Bangor University, Wales, UK and Central South University of Forestry and Technology, Changsha 410004, China; 2 National Engineering Laboratory for Applied Technology in Forestry and Ecology in South China, Central South University of Forestry and Technology, Changsha 410004, China; xchen2@govst.edu (X.C.); t20172350@csuft.edu.cn (Y.L.); wang_changsha@hotmail.com (J.W.); 3 College of Life Science and Technology, Central South University of Forestry and Technology, Changsha 410004, China; 4 College of Arts and Sciences, Governors State University, University Park, IL 60484, USA; 5 Department of Environment and Soil Sciences, University of Lleida, Avinguda Alcalde Rovira Roure 191, 25198 Lleida, Spain; awais.shakoor22@gmail.com; 6 College of Forestry, Fujian Agriculture and Forestry University, Fuzhou 350002, China; haroonrashid383@gmail.com; 7 College of Plant Protection, Fujian Agriculture and Forestry University, Fuzhou 350002, China; Uttam545@gmail.com

**Keywords:** stable isotope, isotopic composition, C and N cycling, vegetation type, soil health

## Abstract

Natural isotopic abundance in soil and foliar can provide integrated information related to the long-term alterations of carbon (C) and nitrogen (N) cycles in forest ecosystems. We evaluated total carbon (TC), total nitrogen (TN), and isotopic natural abundance of C (δ^13^C) and N (δ^15^N) in soil and foliar of coniferous plantation (CPF), natural broadleaved forest (NBF), and mixed forest stands at three different soil depths (i.e., 0–10, 10–20, and 20–40 cm). This study also explored how soil available nutrients are affected by different forest types. Lutou forest research station, located in Hunan Province, central China, was used as the study area. Results demonstrated that the topsoil layer had higher TC and TN content in the mixed forest stand, resulting in a better quality of organic materials in the topsoil layer in the mixed forest than NBF and CPF. In general, soil TC, TN, and δ^15^N varied significantly in different soil depths and forest types. However, the forest type did not exhibit any significant effect on δ^13^C. Overall, soil δ^13^C was significantly enriched in CPF, and δ^15^N values were enriched in mixed forest. Foliar C content varied significantly among forest types, whereas foliar N content was not significantly different. No big differences were observed for foliar δ^15^N and δ^13^C across forest types. However, foliar δ^13^C and δ^15^N were positively related to soil δ^13^C and δ^15^N, respectively. Foliar N, soil and foliar C:N ratio, soil moisture content (SMC), and forest type were observed as the major influential factors affecting isotopic natural abundance, whereas soil pH was not significantly correlated. In addition, forest type change and soil depth increment had a significant effect on soil nutrient availability. In general, soil nutrient availability was higher in mixed forest. Our findings implied that forest type and soil depth alter TC, TN, and soil δ^15^N, whereas δ^13^C was only driven by soil depth. Moreover, plantations led to a decline in soil available nutrient content compared with NBF and mixed forest stands.

## 1. Introduction

Human activities and different natural environmental factors produce abrupt, large scale, irreversible changes and alter forest structure and composition, consequently resulting in the changes of biogeochemical cycles [1,2]. Species composition significantly affects the quality and quantity of carbon (C) and nitrogen (N) input by controlling surface soil and vegetation layer C and N contents and their turnover rates [3,4,5]. N is the essential element in the natural ecosystem, and is a vital limiting resource for plant growth [5]. The C cycle is crucial as it influences soil respiration and the plant photosynthetic process. The ^13^C and ^15^N natural abundance in the soils is a dynamic function of the rate and isotopic composition and the C and N transformations in the forest ecosystem as it provides cohesive insights into C and N cycles [6,7,8]. Over the years, the isotopic composition of C and N in soil has been used to evaluate soil C turnover rates [9]. Despite its significance, the increase in δ^13^C and δ^15^N values in soils remains challenging, especially given the various factors that may discriminate against ^13^C and ^15^N natural abundance within soil profiles [9]. Therefore, ecologists seek numerous pathways to better understand how changes in the forest type impact the ecosystem functioning.

Land is an indispensable natural resource for ecosystem functioning. In general, soil organic carbon usually changes with human activities and land-use change. Litter inputs usually lower the soil δ^13^C and δ^15^N, whereas a higher decomposition rate enhances δ^13^C and δ^15^N [10,11]. However, in stable forest vegetation, soil organic carbon turnover rates do not considerably alter with soil depth profiles [7,12]. In addition, the N cycle processes in forests are impacted by vegetation type change, thus influencing soil δ^15^N [13,14,15,16]. These biogeochemical cycles play an essential role in maintaining the overall ecosystem and soil fertility [17,18].

Plantations are usually fast-growing species with shorter rotation cycles compared to broadleaved natural forests and mixed-species forests. Ever-increasing forest product demand has led to an upsurge in plantation forests worldwide [19]. Since the 1980s, different large-scale afforestation forestry programs have been introduced in China to meet the ever-increasing demand for good quality timber and other forest products, resulting in the conversion of many natural and mixed forests into coniferous monoculture plantations [19,20]. These plantations are man-made and managed differently than natural forest stands. Natural broadleaf forest species are generally more N-rich than coniferous plantations and have less N retention capacity [21]. Moreover, natural forests have typically stable vegetation with natural rotation cycles and less human interference than plantations. Previous studies have described that soil quality indicators and different silvicultural practices such as intercropping/inter cultivation, fallow period, irrigation application techniques, use of organic and inorganic fertilizers, along with human activities, affect greenhouse gas emissions [21,22,23,24], soil C and N storage, loss of inorganic N, soil fertility, and soil structure [25,26]. However, in global analyses, details pertaining to fluctuations in soil and foliar ^13^C and ^15^N natural abundance and their relation with influential factors is scarce regarding the different sub-tropical forest types of eastern Asia. Soil nutrient availability is a crucial indicator of soil fertility [27]. Soil fertility in forest ecosystems can be influenced by numerous factors such as canopy dynamics, litter quality, parent material, land-use history, species composition, environmental factors, and atmospheric deposition [28,29,30]. Thus, this can directly affect the isotopic abundance, forest productivity, and sustainability [31,32,33]. Therefore, studying the δ^15^N, δ^13^C, TC, and TN content in leaves and soil, and variability in soil fertility is expected to provide insights into the species’ resource utilization efficiency in an ecosystem and the consequences of different silvicultural methods for these biogeochemical cycles.

The objectives of this study were: (i) to characterize the variability of soil and foliar total C (TC), total N (TN) content, and ^13^C and ^15^N natural abundance in vertical depth soil profiles in different forest types; and (ii) to analyze the effect of forest type change (different forest types) on soil nutrient availability. Soil and foliar stable isotopic ratios are influenced by atmospheric N_2_ inputs and processes that regulate N cycling and soil organic matter (SOM) transformation. Moreover, different forest management regimes influence the C and N isotopic abundance, for example, clear-cutting practices in plantations increase soil N availability, which can enrich δ^15^N. Therefore, we hypothesize that: (a) TC and TN content and δ^13^C and δ^15^N in soil and foliar would significantly vary in different forest types and at different soil depths; moreover, (b) forest structure and composition would also considerably influence the soil nutrient availability.

## 2. Materials and Methods

### 2.1. Study Area

**Figure 1:**
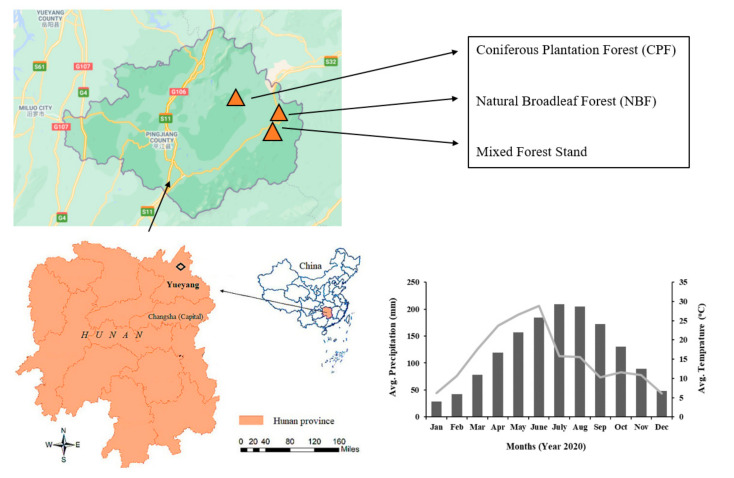
Location and climatic conditions of the study area.

The study was conducted at the Lutou forest ecosystem observation and research Station, Pingjiang County, Yueyang, Hunan Province, China. The geographic coordinates of the research station are 113°51′52″~113°58′24″ E, 28°31′17″~28°38′00″ N (Figure 1). The total area of the research station is 4762 ha. The soil type is the same in all three forest types (Lateritic red soil). The study area is located in the humid subtropical zone. The climate of the study area is warm and humid with abundant rainfall, sufficient sunshine, and four distinct seasons. The mean annual temperature is 17.07 ℃, and the mean annual precipitation is 1312 mm, with an average humidity of 82%.

### 2.2. Forest Types and Species

Natural broadleaved forest (NBF), coniferous plantation forest (CPF), and mixed forest stands were used in the study. NBF and mixed forest stands were adjacent to each other, and the CPF stand was at a 300–400 m (0.19–0.25 mile) distance from the first two forest stands. All three forest stands were present in the same research station. There were no water channels present on the ground in any forest type and watering was natural because of the abundant rainfall. Litterfall biomass and understory vegetation were apparently more in the mixed forest stand. We selected those trees where shrub presence around the canopy projection area of trees was significantly less than the usual shrub presence in the stands. Therefore, the direct influence of the shrub layer would be minimal. Still, these are field conditions; hence indirect effects might be possible. The characteristics of the study area and forest types are shown in Table 1.

Detailed information about (1) NBF, (2) CPF, and (3) mixed forest stands is mentioned below.

1. The mid-subtropical zone of China is covered with natural forests dominated by broadleaf evergreen species, and *Castanopsis eyeri* (Fagaceae) is one of them [34]. *C. eyeri* is a vital part of forest ecosystems and provides services such as water conservation, biodiversity protection, biomass maintenance, and local climate regulation [35]. The *C. eyeri* (NBF) stand in the research station is the largest and most complete community of Castonpsis, and therefore has crucial protection, scientific research, and landscape values. The forest is neat, and the forest canopy undulating. At the Lutou forest farm, it is mainly distributed on mountain slopes. The average slope gradient is 20–25°. The shrub layer includes species such as *Rhododendron simsii*, *Ilex formosana*, *Daphniphyllum oldhami*, *Toxicodendron vernicifluum*, *Cyclobalanopsis glauca*, *Eurya tetragonoclada*, *Symplocos sumuntia*, *Rhododendron simiarum*, *Lindera aggregate*, *Eurya muricat*, *Dendropanax dentiger*, and *Myrica rubra*.
2. Chinese fir (*Cunninghamia lanceolata*) is a typical evergreen coniferous timber tree species. The pure natural forest of *C. lanceolata* is rare to find, and mainly presents as an artificial forest plantation. The CPF stand at Lutou forest station are present on both flat soil and mountain slopes. The average slope gradient is 18–23°. Besides timber production, climate regulation, and soil and water conservation, it also provides biomass energy [21,36]. The CPF stand was established 30 years ago under the Chinese afforestation program for forest area enhancement in 1980–1990. It was established as a plantation forest after clearing the site. The shrub layers’ primary species include *Litsea cubeba*, *Eurya mauricata*, *Rhus chinensis*, *Sapium discolor*, *Rhododendron mariesii*, *Diplospora dubia*, *Ilex chinensis*, and *Rubus lambertianus*.
3. The *P. massoniana* + *C. eyeri* mixed forest is an important forest type at Lutou forest station. In Lutou, it is distributed in the ridges, hillside, and mountain slopes. The average slope gradient was 20–25°. *P. massoniana* is an evergreen coniferous species that has been widely planted in the red soils of southern China since 1980, mainly for soil conservation purposes [37,38]. At the Lutou forest ecosystem observation and research station, the mixed forest stand is formed by planting *P. massoniana* in the natural regenerated *C. eyeri*. The shrub layer primarily comprises *Loropetalum chinensis*, *Rhododendron longipetalon*, *Ilex latifolia*, *Camellia oleifera*, *Phobe hupehensis*, *Ilex ficifolia*, *Eugenia glabra*, *Rubus cranbergii*, *Rata thunbergia*, etc.

### 2.3. Soil and Foliar Sampling

Sampling was carried out in the first week of October 2020. Soil samples were collected from nine selected locations (three locations from each forest type). Three 20 × 20 m plots were established in each forest stand. The soil samples were obtained with a steel soil auger (3.5 cm diameter) up to a depth of 0–10, 10–20, and 20–40 cm soil. After collection, soil samples were appropriately cleaned, and roots/stones were sorted out. Three composite samples per forest type per soil depth were prepared for the analysis. A sufficient number of mature and healthy foliage (5–8 per tree) of each forest type (three plots in each stand) was collected from four to six different individuals (at least 5 m apart from each other) using a long-handled pruner and pooled three composite samples for each forest type. Leaves were placed in a clean, perforated plastic bag [15]. We tried to select the plots with uniform topography to minimize the local terrain impact on trees/vegetation.

Field moist soils were air-dried at room temperature and sieved through a 2 mm mesh size to remove stones, roots, and plant residues. The foliage samples were gently rinsed with distilled water and oven-dried for 72 h at 65 °C. Later, soil and foliar samples were finely ground with a ball mill (JXFSTPRP-64, Jingxin Co. Ltd., China) and used to measure δ^13^C and δ^15^N, C and N content, SOC, and soil nutrient availability analysis [9,15].

### 2.4. Isotopic ^13^C, ^15^N Abundance, and C, N Content Analysis

δ^13^C, δ^15^N, TC, and TN content were measured using an isotope ratio mass spectrometer (IRMS) (IsoPrime 100, Isoprime Ltd., Cheadle, UK), connected to a CN elemental analyzer (Vario MICRO cube, Elementar, Germany). C and N isotopic abundances were calculated as δ^13^C and δ^15^N (‰) using the following formula:

δ^13^C and δ^15^N (‰) = (R_sample_/R_standard_ − 1) × 1000

where R_sample_ is the stable isotopic ratio in the samples and R_standard_ is the ratio in the standard. The Vienna Pee Dee Belemnite (VPDB) was used as a standard for δ^13^C and atmospheric N_2_ was used as the standard for δ^15^N. The precision of isotopic composition was checked using internal standards (i.e., acetanilide, L-histidine, D-glutamic, and glycine) and positive and negative values were observed. Positive delta values indicate that there is a greater percentage of isotope presence relative to the standard whereas negative values indicate a lesser percentage of isotope presence relative to the standard. In general, the analytical precision for δ^13^C and δ^15^N was better than 0.2‰ [9,15].

### 2.5. Determination of Soil Available Nutrients

Available nitrogen (AN) was determined by the Kjeldahl method [39]. Available phosphorus (AP) was determined by the diacid extraction spectrophotometric colorimetry method [40], and available potassium (AK) was determined using a flame photometer method by ammonium acetate extraction [41]. Moreover, soil moisture content (SMC) was calculated based on wet and dry weight, whereas soil pH was determined using a potentiometric method (1:2.5 soil:water). Details about the soil laboratory analysis are also available in our published papers [42,43].

### 2.6. Data Analysis

Two-way analysis of variance (ANOVA) at a 5% probability level was performed to determine the effects of forest types and different soil depth profiles on the natural abundance of C and N isotopes, soil TC and TN content, and soil nutrient availability. One-way ANOVA at 5% probability level was performed to determine the differences between foliar δ^13^C, δ^15^N and, foliar TC and TN content among forest types. A regression analysis was conducted to analyze the foliar and soil isotopic abundance relationship. The Pearson correlation test was performed to observe the relationship between isotopic dynamics and influential factors. Means that exhibited significant differences were compared using post-hoc Tukey’s HSD significance test. All statistical analyses were performed using the SPSS Statistical Package (SPSS 17.0, Chicago, IL, USA).

## 3. Results

### 3.1. Soil and Foliar TC, TN Content, and C:N Ratio

Two-way ANOVA revealed that soil TC, TN content, and C:N ratio varied significantly among forest types (*p* < 0.05) and soil depths (*p* < 0.001) (Table 2 and Table A1). Soil TN content ranged from 0.56 g·kg^−1^ to 3.33 g·kg^−1^, while the soil TC content varied from 1.69 g·kg^−1^ to 46.88 g·kg^−1^. Moreover, the soil C:N ratio ranged from 3.01 to 14.06 (Table 2). Soil TN, TC, and C:N ratio decreased as the soil depth increased among all forest types. In the topmost soil layer, TC, TN, and soil C:N ratio was higher in the mixed forest stand, while for the remaining two soil layers, it was greater in CPF (Table 2).

In terms of foliage values, one-way ANOVA showed that foliar C content varied significantly among forest types (*p* < 0.05), whereas foliar N content and foliar C:N ratio were not significantly different (*p* = 0.242 and *p* = 0.155, respectively) (Table 2).

### 3.2. C^13^ and ^15^N Natural Abundance

**Figure 2:**
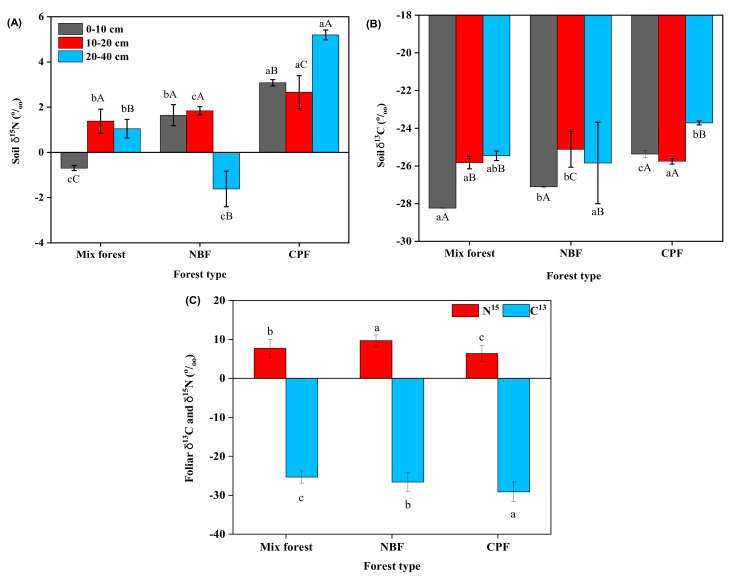
The natural abundance of (**A**) soil ^15^N (‰) and (**B**) soil ^13^C (‰) at different soil depths and (**C**) foliar ^15^N (‰) and ^13^C (‰) in different forest types. Values are means ±SE. The different small letters represent the significant differences between forest types, and different capital letters represent the significant difference between different soil depths *p* ≤ 0.05.

Two-way ANOVA depicted that soil δ^15^N varied significantly among forest types (*p* > 0.001) and soil depths (*p* = 0.048). However, forest type (*p* = 0.078) and interaction of forest type × soil depth (*p* = 0.329) had no significant effect on soil δ^13^C, whereas soil δ^13^C varied significantly with soil depth (*p* = 0.028) (Figure 2 and Table A1). A positive correlation was present between soil δ^15^N-soil depth (*r* = 0.606) and soil δ^13^C-soil depth (*r* = 0.470). Overall, soil δ^15^N values ranged from −1.61‰ to 5.19‰, and soil δ^13^C values ranged from −28.23% to −25.00% (Figure 2).

One-way ANOVA illustrated that forest type had a considerable effect on foliar δ^13^C (*p* = 0.03) and foliar δ^15^N (*p* = 0.047). Foliar δ^15^N values ranged from 6.39‰ to 8.90‰, and foliar δ^13^C values ranged from −25.4‰ to −29.7‰. Foliar δ^15^N was enriched in the NBF stand, and δ^13^C was less negative in the mixed forest stand (Figure 2).

### 3.3. Relationship between Soil, Foliar Isotopic Abundance, and C:N Ratio

**Figure 3:**
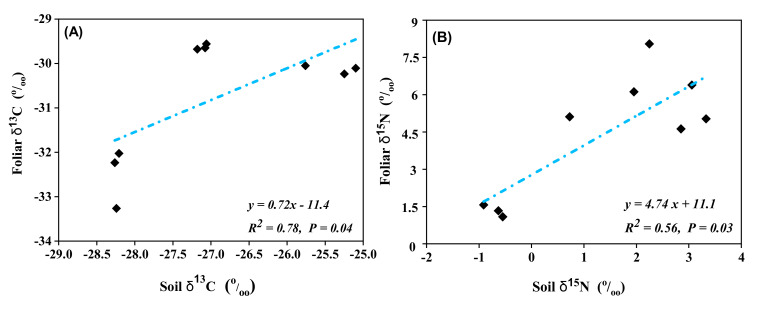
Linear relationships between (**A**) soil and foliar δ^13^C and (**B**) soil and foliar δ^15^N (‰) across three forest types at a 0–40 cm soil depth.

A strong positive linear relationship was observed between soil and foliar δ^13^C (*r* = 0.78, *p* = 0.04) and soil and foliar δ^15^N (*r* = 0.56, *p*= 0.03) across forest types and soil depths (Figure 3A,B).

### 3.4. Correlation between Isotopic Abundance and Potentially Influential Factors

Soil δ^13^C was positively correlated to SMC and foliar C:N ratio, while negatively correlated to soil and foliar TN and soil TC whereas soil δ^15^N was positively related to soil C:N ratio, foliar C:N ratio, and negatively correlated to SMC, foliar TC, and TN content (Table 3).

Foliar δ^13^C was positively and negatively correlated to soil BD and foliar C:N ratio, respectively, whereas foliar δ^15^N was positively related to foliar TC and TN content, and negatively correlated to foliar C:N ratio and SMC (Table 3).

### 3.5. Soil Nutrient Availability

Two-way ANOVA illustrated that AN, AP, and AK varied significantly in different forest types and soil depths (*p* < 0.05) (Table 4 and Table A1). In the 0–10 cm and 20–40 cm soil layers, AN content (177 mg·kg^−1^ and 109 mg·kg^−1^) was observed to be higher in the mixed forest; in 10–20 cm, it was higher in the NBF (155 mg·kg^−1^). AN content decreased as the soil depth increased in the mixed forest and NBF. AP content ranged from 29 mg·kg^−1^ to 82 mg·kg^−1^. In the 0–10 cm soil layer, the highest AP content was observed in CPF (71 mg·kg^−1^), while in the remaining two layers (10–20 and 20–40 cm), the maximum content was measured in the mixed forest. Among all forest types and soil depths, AK ranged from 12 g·kg^−1^ to 147 g·kg^−1^. In the topmost soil layer, it was higher in the mixed forest stand. In both the 10–20 cm and 20–40 cm layers, it was higher in NBF. Generally, soil nutrient content decreased as the soil depth increased across all forest types (Table 4).

## 4. Discussion

Among all forest types, soil TN content decreased as the soil depth increased. In the topsoil layer, both TC and TN contents were enriched in the mixed forest. It indicates that litter input was higher in the mixed forest due to mixed species composition and more understory vegetation, which increased soil organic matter (SOM) more than both NBF and CPF stands. Since soil organic carbon changes with the vertical soil depth profiles, soil depth is widely used as a SOM decomposition rate indicator [44,45,46]. It also points toward the better leaf litter quality in mixed forest with higher N release potential during decomposition. This study observed a considerable difference in soil and foliar TC, TN content, and C:N ratio among the NBF, CPF, and mixed forest. Tree species vary in their nutrient transformation potential with changing forest structure and canopy dynamics. These factors influence the various biogeochemical cycles [12,26,42]. Species autecology affects the decomposition and the turnover rate of C and N dynamics [47]. Neirynck et al. [48] mentioned that different tree species such as *Acer psedoplatanus*, *Fraxinus excelsior*, and *Tilia platyphyllos* significantly affect the C:N ratio and it was related mainly to litter quality. Lovett et al. [49] also revealed that forest structure alters the C and N dynamics because species composition affects nutrient cycling under their different canopy structures, which can substantially influence the forest ecosystems’ overall nutrient turnover rates [50,51].

In this study, a higher abundance of soil ^15^N and ^13^C was observed in CPF. Enriched soil δ^15^N and δ^13^C in CFP could be due to more intensive forest management practices than that in the NBF and mixed forest stands because the plantations are artificially managed forests with added activities of humans and livestock. Pardo et al. [13] and Yang et al. [52] also reported that different silvicultural techniques in plantations such as clear-cutting, slash burning, site preparation, thinning, and pruning could enrich the soil δ^13^C and δ^15^N in plantation forests because of higher soil C and N loss such as enhanced nitrification followed by nitrate loss, which could have a direct impact on isotopic abundance. Rehman et al. [53] reported that various forest protection activities could influence stable isotopic abundance and C:N ratios. Moreover, it is generally believed that multiple factors such as light availability, litter input, water use efficiency, soil physical, chemical, and biological properties altered by the forest types may affect natural isotopic abundance in forest ecosystems [50,51].

Forest type and soil depth significantly impacted soil δ^15^N, whereas soil δ^13^C was only impacted by soil depth; moreover, both the soil δ^13^C and δ^15^N were enriched in the topmost soil layer. Higher isotopic values in the upper soil layer can be due to higher SOC. Ellert and Janzen [54] reported higher C content in the uppermost layer, providing ample SOM that strongly influences the δ^13^C in the topsoil, whereas lesser C content in the deeper layers determines the lesser δ^13^C values. Ngaba et al. [9] also reported that soil depth increment significantly impacted the C and N isotopic abundance. Forest type had a significant effect on foliar C (highest in CPF) and N (highest in NBF) abundance. Usually, foliar δ^15^N values increased under N-rich conditions; in our study, higher foliar and soil δ^15^N were observed in CPF. On average, foliar and soil δ^15^N patterns in sub-tropical forests are higher than other forest types because of the gaseous N losses related to microbial activities. Our study showed foliar C and N positively correlated with the soil isotopic values; this indicates that foliar isotopic values of different species are consistent concerning soil values.

When we studied the correlation of potentially influential factors concerning δ^13^C and δ^15^N in soil and foliage, we observed that SMC, foliar TN, and foliar C:N ratio was usually significantly related to soil foliar ^13^C and ^15^N natural abundance, either positively or negatively. These factors can affect biogeochemical processes such as N mineralization, nitrification/denitrification, soil respiration, root dynamics, thus altering the rate of δ^15^N [55,56]. Moisture content is a vital factor influencing the nutrient content and δ^13^C of the plant and soil. Philips et al. [57] stated that the δ^13^C depends on moisture conditions, either caused by low atmospheric humidity, less precipitation, or low soil moisture. Moreover, drought can also potentially influence the C fluxes and soil δ^13^C [58]. C isotopic abundance primarily depends on the water use efficiency; therefore, soil water resources can affect the isotopic abundance [59]. Ngaba et al. [9] also stated mean annual precipitation and land-use as the significant factor altering soil δ^13^C. While analyzing the ^15^N abundance between the north and south slopes of forests, Chen et al. [5] also reported soil moisture content, leaf N content, and leaf C:N as the significant factors related to isotopic abundance. This inferred that water retention capacity should be the critical factor concerning the isotopic abundance of plant tissues.

In our study, a significant difference was observed in variability in macronutrient availability among all forest types. Generally, AN and AK were higher in mixed forest stand and AP in CPF. Plantation forests mostly have fast-growing species with successive short rotations; hence, the fast growth demands more extensive nutrient availability, which causes an imbalance in soils under plantations compared to mixed-species forest. Kooch et al. [47] reported the decline in soil fertility with successive planting of fast-growing monoculture species at the same site. The decline in soil available nutrients with soil depth increase could be due to a large amount of plant litter deposition in the topsoil layer as its decomposition results in a higher accumulation of soil nutrients in the uppermost soil than the lower soil layers. Farooq et al. [43] cited that species structure influences the soil properties, which is more noticeable at the upper layer of soil. Our study is also in line with Selvaraj et al. [60], who stated that both available and soil total nutrient content decreased with the soil depth, irrespective of stand age; moreover, Groppo et al. [61] and Breulmann et al. [62] also supported this phenomenon regardless of species and land use. However, the relative importance of the various soil-forming factors always remains debated [28,29,30,60]. Soil conditions solely do not affect directly, but with an interplay of other related factors such as the autecology of tree species, litter quality, parent material, soil community effects, human activities, and local climatic conditions [3,63,64,65]. These impacts are likely to have significant consequences for belowground communities [1]. Similarly, different land uses cannot directly affect soil fertility, but it can influence through indirect effects such as organic/inorganic amendments [66,67,68], various stresses for plant and nutrient availability [69,70], different planting materials [71,72], and atmospheric deposition levels and turnover rates [73].

## 5. Conclusions

This study explored how δ^13^C, δ^15^N, TC, and TN content of soil and foliage and soil nutrient availability is affected by different forest types. Forest type and soil depth significantly affected the soil δ^15^N, while forest type effect on soil δ^13^C was not significant. Significant changes in soil δ^15^N among different forest types indicated that ^15^N signatures might dominate the processes of N cycling in different forest types. Across forest types and soil depths, the observed δ^13^C and δ^15^N variations were attributed to SMC, soil TC, soil TN, and soil C:N ratio. Variations in foliar δ^13^C were accredited to differences in soil BD and foliar C:N ratio, whereas variation in foliar δ^15^N was accredited to SMC, foliar TN, and TC. Our study also highlights that, on average, available forms of soil nutrients were less in monoculture Chinese fir stands (CPF) than NBF and mixed forests. This difference was possibly caused by higher litter biomass accumulation from broadleaved and mixed-species litter compared to CPF. Thus, the presence of broadleaved species within a monoculture stand along with other management practices such as the introduction of multi-layering and multi-aged plantations, avoiding clear-cutting practice, and burying the cutting leftover rather than burning could improve soil health, sustainable management, and production, which would enhance both economic and environmental aspects to the forest industry. Overall, our results highlight the importance of forest types and composition when studying biogeochemical cycling and its response in different terrestrial ecosystems. Moreover, this study will also help to further understand the role of C and N cycles concerning soil productivity.

## Figures and Tables

**Table 1:** Characteristics of the study area and selected forest types. Values are mean ±SE. Different small letters represent the significant difference among forest types at *p* ≤ 0.05.

	Mixed Forest	NBF ^a^	CPF
Species	*C. eyeri* + *P. massoniana* ^b^	*C. eyeri*	*C. lanceolata*
Elevation	777 m (a.s.l)	800 m (a.s.l)	427 m (a.s.l)
Soil pH (0–40 cm)	4.60 ± 0.06 ^b^	4.40 ± 0.15 ^c^	4.81 ± 0.02 ^a^
Soil BD (0–40 cm) ^c^	1.28 ± 0.03 ^a^	1.12 ± 0.08 ^c^	1.25 ± 0.03 ^b^
SMC (%) (0–40 cm)	16.3 ^a^	11.63 ^b^	10.48 ^b^
Forest age	30–60 years	60 years	30 years

^a^ NBF is natural broadleaf forest; CPF is coniferous plantations forest. ^b^ *C. eyeri*, *Castanopsis eyeri*; *C. lanceolata*, *Cunninghamia lanceolata*; *P. massoniana* is *Pinus massoniana.* ^c^ BD, soil bulk density; SMC, soil moisture content.; a.s.l, above sea level.

**Table 2:** Soil and foliar TC, TN content, and C:N ratio in different forest types at different soil depths.

	Elements	Depth	Mix Forest	NBF	CPF
Soil	TN ^a^ (g·kg^−1^)	0–10	3.33 ± 0.03 ^aA^	1.56 ± 0.01 ^cA^	2.10 ± 0.02 ^bA^
		10–20	1.12 ± 0.03 ^bB^	0.77 ± 0.01 ^cB^	1.83 ± 0.01 ^aB^
		20–40	0.79 ± 0.04 ^bC^	0.56 ± 0.01 ^cC^	1.19 ± 0.01 ^aC^
	TC (g·kg^−1^)	0–10	46.88 ± 0.44 ^aA^	16.63 ± 0.24 ^cA^	26.02 ± 0.28 ^bA^
		10–20	10.19 ± 0.07 ^bB^	4.17 ± 0.09 ^cB^	19.40 ± 0.06 ^aB^
		20–40	5.33 ± 0.06 ^bC^	1.69 ± 0.03 ^cC^	8.91 ± 0.09 ^aC^
	C:N	0–10	14.06 ± 0.10 ^aA^	10.65 ± 0.23 ^bA^	12.39 ± 0.12 ^aA^
		10–20	9.07 ± 0.25 ^bB^	5.40 ± 0.06 ^cB^	10.62 ± 0.12 ^cB^
		20–40	6.73 ± 0.32 ^aC^	3.01 ± 0.07 ^bC^	7.48 ± 0.15 ^aC^
Foliar	TN (g·kg^−1^)	-	27.28 ± 1.96 ^ab^	28.92 ± 1.43 ^a^	26.40 ± 0.68 ^b^
	TC (g·kg^−1^)	-	482.23 ± 3.77 ^b^	495.72 ± 0.27^a^	475.30 ± 0.42 ^c^
	C:N	-	15.33 ± 1.56 ^a^	17.21 ± 0.83 ^a^	18.02 ± 0.48 ^a^

Note: Values are means ±SE. The different small letter represents the significant differences between forest types and different capital letters between different vertical soil depths at *p* ≤ 0.05. **^a^** TN, total nitrogen; TC, total carbon.

**Table 3:** Pearson correlation between soil and foliar δ^13^C and δ^15^N and potentially influential factors across forest types and soil depths.

	Foliar δ^13^C		Soil δ^13^C			Foliar δ^15^N		Soil δ^15^N	
	*r*	*p*	*r*	*p*		*r*	*p*	*r*	*p*
Foliar TN ^a^	0.220	0.263	−0.526 **	0.001	Foliar TN	0.923 **	0.001	−0.414 *	0.032
Soil TN	0.118	0.551	−0.470 *	0.015	Soil TN	−0.152	0.448	−0.058	0.774
Foliar TC	0.047	0.81	−0.190	0.346	Foliar TC	0.395 *	0.041	−0.526 **	0.004
Soil TC	0.167	0.403	−0.504 **	0.012	Soil TC	−0.124	0.536	−0.126	0.531
Foliar C:N	−0.724 **	0.001	0.551 **	0.018	Foliar C:N	−0.780 **	0.001	0.404 *	0.036
Soil C:N	0.088	0.665	−0.406 *	0.038	Soil C:N	−0.194 *	0.033	0.141 *	0.048
Soil pH	−0.198	0.324	0.107	0.591	Soil pH	−0.285	0.149	0.194	0.332
BD	0.567 **	0.002	−0.043	0.862	BD	−0.098	0.626	0.076	0.707
SMC	−0.431	0.130	0.359 **	0.015	SMC	−0.321 **	0.001	−0.478 **	0.001
Forest type	−0.261	0.186	0.267	0.179	Forest type	−0.463 *	0.015	0.616 **	0.001
Soil depth	0.005	1.02	0.470 *	0.013	Soil depth	0.005	1.01	0.040 *	0.842

Note: Correlation was significant at the 0.05 * and 0.01 ** level (2-tailed). **^a^** TN, total nitrogen; TC, total carbon; BD, soil bulk density; SMC, soil moisture content.

**Table 4:** Available soil nutrients in different forest types at different soil depths.

Elements	Depth	Mix Forest	NBF	CPF
Available N (mg·kg^−1^)	0–10	177 ± 2.06 ^aA^	167 ± 2.04 ^bA^	155 ± 2.2 ^cA^
	10–20	142 ± 2.49 ^bB^	155 ± 2.45 ^aB^	137 ± 1.58 ^cB^
	20–40	109 ± 1.0 ^aC^	105 ± 3.54 ^bC^	98 ± 1.15 ^cC^
Available P (mg·kg^−1^)	0–10	62 ± 2.42 ^bB^	55 ± 0.75 ^cA^	71 ± 0.69 ^aA^
	10–20	82 ± 2.46 ^aA^	54 ± 0.47 ^bA^	36 ± 1.22 ^cB^
	20–40	60 ± 2.59 ^aB^	38 ± 0.29 ^bB^	29 ± 0.15 ^cC^
Available K (mg·kg^−1^)	0–10	147 ± 0.37 ^aA^	104 ± 1.94 ^bB^	91 ± 0.81 ^cA^
	10–20	41 ± 1.02 ^bB^	119 ± 1.71 ^aA^	22 ± 0.85 ^cB^
	20–40	22 ± 0.07 ^bC^	41 ± 0.92 ^aC^	12 ± 0.21 ^cC^

Note: Values are means ±SE. The different small letters represent the significant differences between forest types, and different capital letters represent the significant difference between soil depths at *p* < 0.05.

## Data Availability

All the available data have been presented in the manuscript.

## Abbreviations

NBF—natural broadleaved forest; CPF—coniferous plantations forest; SMC—soil moisture content; BD—soil bulk density; SOM—soil organic matter; MAT—mean annual temperature; MAP—mean annual precipitation; AN—available nitrogen; AP—available phosphorus; AK—available potassium.

## Appendix A

**Table A1:** Two-way ANOVA results (*p* values) for the studied soil variables at *p* ≤ 0.05.

Variable	Forest Type	Soil Depth	Forest Type × Soil Depth
TN	<0.05	<0.001	0.02
TC	0.04	<0.001	0.04
δ^15^N	<0.001	0.048	<0.001
δ^13^C	0.078	0.028	0.329
C:N	<0.001	<0.001	0.04
SOC	0.01	0.01	0.03
AN	0.04	<0.001	0.02
AP	0.02	0.03	<0.001
AK	0.05	0.05	0.02
